# Synergistic Effect of Compost and Subsurface Water Retention Technology on Optimizing Soil Properties and Argan (*Argania spinosa* L. Skeels) Performances Under Field Conditions

**DOI:** 10.3390/plants15030365

**Published:** 2026-01-24

**Authors:** Boujemaa Fassih, Mohamed Ait-El-Mokhtar, Aicha Nait Douch, Abderrahim Boutasknit, Redouane Ouhaddou, Chayma Ikan, Zoulfa Roussi, Raja Ben-Laouane, Badia Aganchich, Said Wahbi

**Affiliations:** 1 Centre d’Agrobiotechnologie et Bioingénierie, Unité de Recherche Labellisée CNRST (Centre AgroBiotech-URL-CNRST-05), Cadi Ayyad University, Marrakesh 40000, Morocco; 2 Laboratory of Biotechnology, Agri-Food, Materials, and Environment (LBAME), Department of Biology, Faculty of Science and Techniques Mohammedia, Hassan II University of Casablanca, Mohammedia 28800, Morocco; 3 Laboratory of Biology, Geosciences, Physics and Environment, Pluridisciplinary Faculty of Nador, University Mohamed Premier, B.P. 300, Seloune, Nador 62700, Morocco; 4 Agricultural Innovation and Technology Transfer Center, College of Agriculture and Environmental Sciences, Mohammed VI Polytechnic University, Lot 660, Hay Moulay Rachid, Ben Guerir 43150, Morocco; 5 Laboratory of Environment and Health, Department of Biology, Faculty of Science and Techniques, Errachidia 52000, Morocco

**Keywords:** argan, water management, soil amendment, climate change resilience, plant physiology, antioxidant activity

## Abstract

*Argania spinosa* L. Skeels is an ecological pillar of the arid zones of South-West Morocco, currently threatened by the drastic climate change. This study investigates the effect of the combined application of compost (C) and subsurface water retention technology (SWRT) on field performances of one-(1Y) and two-year-old (2Y) argan seedlings. A randomized field trial was performed with four treatments: Control, C, SWRT, and C + SWRT. We evaluated soil properties, growth, and physiology, alongside biochemical parameters including stress markers, compatible solutes, antioxidant enzyme activities, and secondary metabolites. The results reveal the significant effect of C and/or SWRT on argan seedlings performances, particularly in 1Y subjects. The C + SWRT strongly stimulated stem elongation (246% vs. 163%), stomatal conductance (75% vs. 99%), photosynthetic efficiency (18% vs. 11%), and chlorophyll a content (80% vs. 65%) in 1Y and 2Y seedlings, respectively, compared to their corresponding controls. Under the same treatment, malondialdehyde levels were significantly reduced by 37% in 1Y seedlings and 23% in 2Y seedlings. In addition, catalase activity and soluble sugar, protein, and polyphenol content increased by 38, 43, 26, and 21%, respectively, in the younger seedlings and by 53, 51, 18, and 19%, respectively, in the elder seedlings. In terms of soil health, C + SWRT significantly enhanced total organic carbon and matter, available phosphorus, and reduced electrical conductivity. In summary, the C + SWRT application significantly improved argan plant performances, with a particularly marked effect on 1Y seedlings, which makes this combination an alternative solution to enhance the resilience of the argan tree in the era of climate change and promote the success of the reforestation program.

## 1. Introduction

In the Mediterranean ecosystem, plants are frequently exposed to prolonged and severe drought stress [1]. Among these, the argan tree *Argania spinosa* (L.) Skeels, an endemic species in Morocco, has shown exceptional adaptability to such harsh conditions [2]. This tree is a keystone species of profound ecological, economic, and cultural importance and is adapted to arid and semi-arid climates where it plays a crucial role in soil erosion prevention, biodiversity conservation, and local ecosystem support [3]. The local communities benefit from the high-value products of the tree, in particular argan oil, a world-renowned product for use in cosmetics, medicine, and gourmet markets. Argan oil has become one of the most expensive oils in the world due to growing demand at both national and international levels. Due to the multiple uses of argan trees, the growing needs, and the severity of the climate, areas covered by argan trees are shrinking at an average rate of 2000 ha/year [4]. Furthermore, the natural regeneration of the argan forest is currently facing a critical decline driven by the synergistic effects of environmental stressors and anthropogenic pressures. A major factor is climate change, as increasing aridity and shifting climate patterns are significantly contracting the potential distribution area of *A. spinosa*, thereby limiting its regeneration performances [5]. In addition to climate change impact, overgrazing poses a severe threat to ecosystem stability; intensive browsing induces physiological stress by altering photosynthetic behavior [6], while simultaneously compromising the structural integrity and development of argan populations, ultimately exacerbating the degradation of these woodlands [7].

In the late 1990s, the Moroccan Forestry Department launched a large-scale reforestation program based on assisted natural regeneration, aiming to conserve the argan tree and reverse its ongoing decline. However, establishing successful argan plantations remains a major challenge, and many reforestation projects have ended in failure [8]. In response to these challenges, the development of sustainable and adaptive reforestation practices is essential to ensure the long-term survival and establishment of argan seedlings in degraded and arid ecosystems. Recently, a comprehensive bibliometric analysis of *Argania spinosa* L. Skeels research highlighted the urgent need to shift from simple conservation to strategic, climate-resilient ecosystem management to ensure the sustainability of these forests [9]. One promising approach is the use of compost, which significantly improves soil fertility and structure, thereby increasing plant growth and enhancing resilience [10,11]. Beyond nutrient enrichment, compost increases the soil’s water retention capacity—an essential feature for seedling establishment in water-limited environments [12,13]. Complementing this strategy, subsurface water retention technology (SWRT) has emerged as an innovative and effective solution for improving water use efficiency in arid and semi-arid regions. This technology is based on the installation of an impermeable barrier beneath the root zone, which limits deep percolation losses and retains water within the active rooting layer. By modifying water movement, SWRT increases soil moisture residence time in the rhizosphere, enhances root access to water, and improves plant water status under drought conditions. Through these mechanisms, SWRT helps sustain plant development under drought stress and contributes to more resilient reforestation systems [14,15,16].

Although compost alone is beneficial for improving soil fertility and organic matter content, notably through the supply of nutrients, stimulation of microbial activity, and enhancement of cation exchange capacity [17], its potential to enhance water retention especially in coarse-textured or degraded soils remains limited. While compost can increase aggregate formation and short-term moisture retention by adding organic binding agents and microbial by-products [17], its effects on soil structure and porosity tend to be moderate and often diminish over time. As a result, compost may not effectively prevent deep percolation or ensure long-term water availability in the rhizosphere. In arid environments, where prolonged drought is common, compost alone often fails to maintain sufficient moisture within the root zone. To address these limitations, integrating compost with SWRT presents a more effective strategy. This integration not only enhances the soil’s water-holding capacity but also improves water use efficiency, thus reinforcing plant resilience under water-limited conditions. In this context, the age of seedlings at transplantation becomes a critical factor influencing establishment success [18,19].

In species adapted to arid climates, interaction between transplant age and soil management practices can significantly influence establishment success and yield [20]. Consequently, a holistic approach that combines optimized soil water management through compost–SWRT integration with appropriate transplant age is essential for maximizing argan seedling performance, and long-term productivity. However, despite its relevance, the combined effects of transplant age and Compost + SWRT (C + SWRT) application on the growth and physiology of argan seedlings remains underexplored and warrants further investigation.

In this context, the primary objective of our study is to evaluate the individual and combined effects of SWRT and compost on soil fertility as well as the performances of one- (1Y) and two-year-old (2Y) argan seedlings under field conditions. Specifically, we aim to (i) evaluate how compost and SWRT, applied alone or in combination, influence soil fertility, (ii) determine their effects on seedling growth, physiological, and biochemical performance, and (iii) examine whether transplant age modulates seedling responses to these soil management strategies. We hypothesize that the combined compost-SWRT treatment will exert synergistic effects on promoting argan performances, with younger seedlings exhibiting greater responsiveness due to their higher sensitivity to soil water availability. The findings will contribute to developing science-based strategies for sustainable argan reforestation and arid-land restoration particularly in the face of increasing climate change challenges.

## 2. Results

### 2.1. Effect of Compost and/or SWRT Technology on Soil Physicochemical Parameters

The soil physicochemical results (Table 1) show that the pH remained alkaline overall in all treatments, with values close to 8. No significant variation in pH was observed according to the treatments applied or the age of the argan seedlings. The application of SWRT significantly decreased levels of soil available phosphorus (AP) significantly increased in soils amended with compost, particularly when combined with SWRT, compared with unamended soils, for both age groups of seedlings. Soil nitrogen measurements showed a significant increase, whatever the age and treatment factor. For total organic carbon (TOC) and organic matter (OM), the C + SWRT exhibited significantly higher values in both argan age groups compared to the control.

### 2.2. Effects of Compost and SWRT on Argan Seedlings Growth

**Figure 1:**
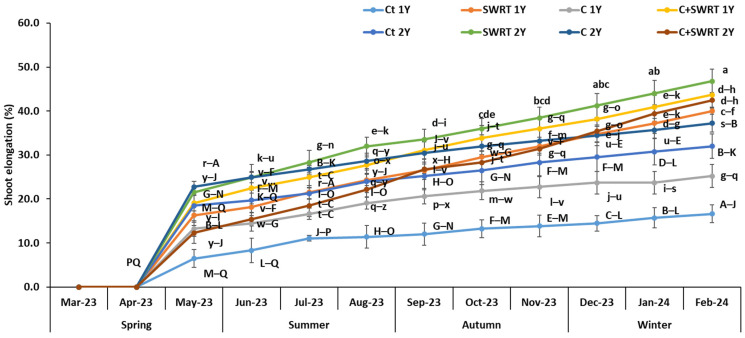
Evolution of the effect of compost and/or SWRT application on argan shoot elongation of one- and two-year-old argan seedlings. Ct: control; SWRT: with SWRT; C: compost; C + SWRT: combined compost and SWRT. Different letters indicate significant differences (*p* < 0.05) according to Tukey’s test.

Results shown in Figure 1 illustrate the seasonal variation in shoot elongation of 1Y and 2Y argan seedlings treated with SWRT and/or compost. The application of SWRT alone or combined with compost (C + SWRT) resulted in a significant increase in stem elongation of 1Y seedlings by 169 and 246%, respectively, compared to the control seedlings (1Y). Similarly, 2Y seedlings treated with SWRT alone or in combination with compost (C + SWRT) showed significantly higher growth than the control seedlings, with increases of 141 and 163% compared to the control seedlings (2Y). In addition, the application of compost alone (C) induced an increase of 102% in 1Y seedlings and 52% in 2Y seedlings. Compared to the 2Y seedlings, 1Y seedlings performed better, especially when they were treated with C + SWRT.

### 2.3. Effects of Compost and SWRT on Argan Seedlings Physiological Responses

#### 2.3.1. Stomatal Conductance

**Figure 2:**
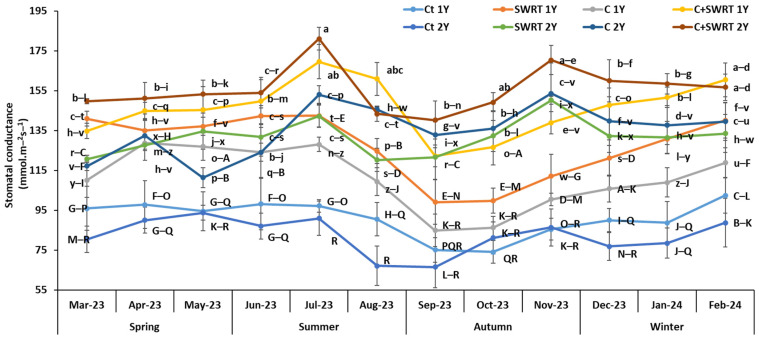
Evolution of the effect of compost and/or SWRT application on stomatal conductance of one- and two-year-old argan seedlings. Ct: control; SWRT: with SWRT; C: compost; C + SWRT: combined compost and SWRT. Different letters indicate significant differences (*p* < 0.05) according to Tukey’s test.

Monthly monitoring of gs in 1Y and 2Y argan seedlings showed significant variations depending on the applied treatments (Figure 2). The stomatal conductance (gs) of argan seedlings generally varied between 60 and 180 nmol.m^−2^s^−2^. The combined C + SWRT treatment proved to be the most effective in improving this physiological parameter, particularly during the summer season. In Jully, seedlings treated with C + SWRT showed a 99% increase in gs for 2Y seedlings and a 75% increase for 1Y seedlings, compared to their respective controls. In contrast, the lowest values were registered in September. The separate application of C and SWRT also led to a noticeable improvement in gs during the Summer, with increases of 31 and 46%, respectively, in 1Y seedlings, compared to the controls from the same season. In 2Y seedlings, the most pronounced effects of separate application of C and SWRT were observed in Jully with significant increases of 78 and 74%, respectively, compared to the controls. Generally, this parameter was particularly improved in the 2Y seedlings treated with C and/or SWRT compared to the 1Y seedlings during the experiment duration.

#### 2.3.2. Photosynthetic Efficiency

**Figure 3:**
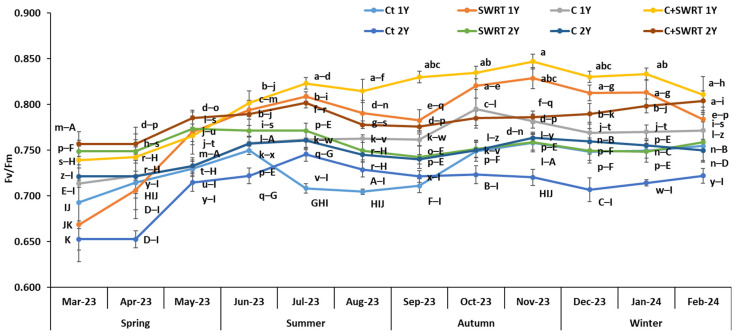
Evolution of the effect of compost and/or SWRT application on the maximum quantum efficiency of PSII (Fv/Fm) of one- and two-year-old argan seedlings. Ct: control; SWRT: with SWRT; C: compost; C + SWRT: combined compost and SWRT. Different letters indicate significant differences (*p* < 0.05) according to Tukey’s test.

Data shown in Figure 3 illustrate the seasonal variation in the maximum quantum efficiency of PSII (Fv/Fm) in 1Y and 2Y argan seedlings subjected to different treatments. The application of SWRT alone or in combination with C significantly improved Fv/Fm by 9 and 11% for 2Y seedlings, and by 5 and 6% for 1Y seedlings, respectively, during spring compared to their control counterparts. However, during the summer season, 1Y seedlings demonstrated significantly greater improvements compared to 2Y seedlings, with increases of 18, 14, and 7% for the C + SWRT, SWRT, and C treatments, respectively. After slight declines in autumn, both age groups exhibited a recovery, with 1Y seedlings showing significantly higher improvements in Fv/Fm when treated with C + SWRT or SWRT alone compared to 2Y argan seedlings.

#### 2.3.3. Photosynthetic Pigments Content

**Figure 4:**
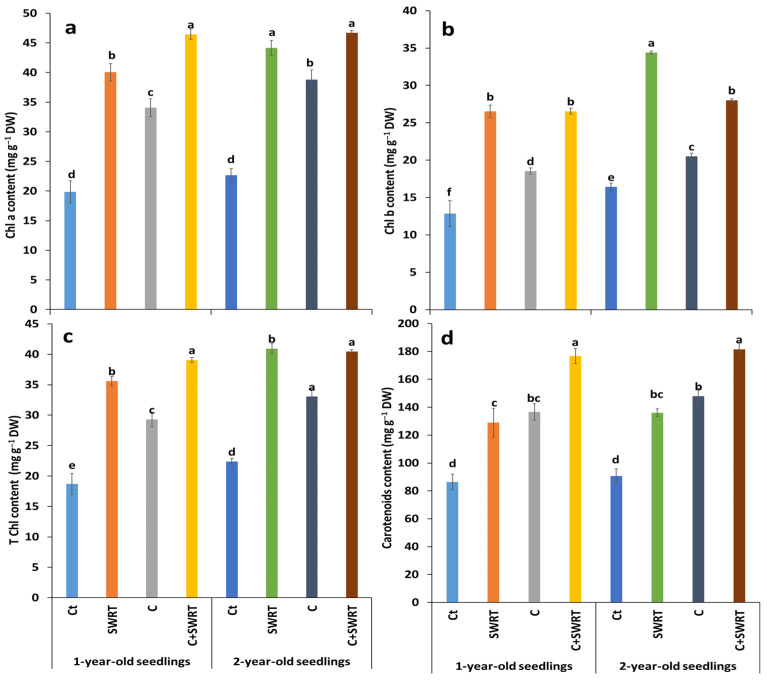
Effect of compost and/or SWRT application on chlorophyll a (Chl a, (**a**)), chlorophyll b (Chl b, (**b**)), total chlorophyll (T Chl, (**c**)), and carotenoid (**d**) content in one- and two-year-old argan seedlings. Ct: control; SWRT: with SWRT; C: compost; C + SWRT: combined compost and SWRT. Different letters indicate significant differences (*p* < 0.05) according to Tukey’s test.

The results presented in Figure 4, demonstrate that the combined treatment C + SWRT significantly enhanced photosynthetic pigments content in both 1Y and 2Y seedlings compared to the controls. Chlorophyll a (Chl a), chlorophyll b (Chl b), and total chlorophyll (TChl) were significantly improved by the combined application of C and SWRT, particularly in 1Y argan seedlings, with respective increases of 80, 57, and 69% compared to the controls. In 2Y seedlings, this combination also led to a notable improvement in pigment content (64, 35, and 50% for Chl a, Chl b and TChl, respectively). In addition, the separate application of C and SWRT also had a positive effect, albeit more moderate. In 1Y seedlings, Chl a increased by 50% with C and 64% with SWRT, while Chl b increased by 25% with C and 68% with SWRT. The content of TChl increased by 38% with C and 66% with SWRT. In 2Y seedlings, the relative gains were 24 and 28% for Chl b and 26 and 34% for TChl under C and SWRT, respectively, compared to the controls. Carotenoid content followed a similar trend. Significant increases were observed under the combined application of C + SWRT, with a 57% increase in 1Y seedlings and a 60% increase in 2Y seedlings. The application of C also improved this content, with increases of 38% in 1Y seedlings and 40% in 2Y seedlings, again compared to the controls. Consequently, it should be noted that the SWRT-based treatments further improved the photosynthetic pigment composition in 2Y seedlings than in 1Y seedlings.

### 2.4. Effects of Compost and SWRT on Argan Seedlings Biochemical Responses

#### 2.4.1. Hydrogen Peroxide and MDA Content

**Figure 5:**
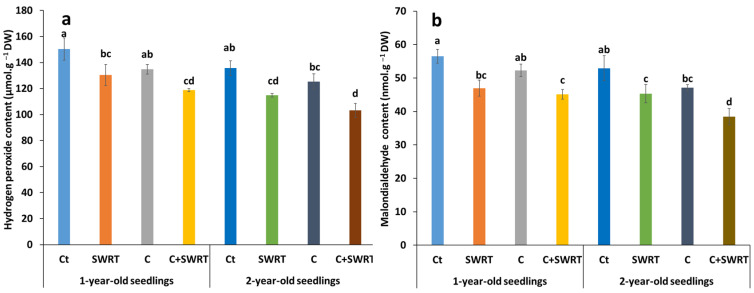
Effect of compost and/or SWRT application on hydrogen peroxide (H_2_O_2_, (**a**)) and malondialdehyde (MDA, (**b**)) content in one- and two-year-old argan seedlings. Ct: control; SWRT: with SWRT; C: compost; C + SWRT: combined compost and SWRT. Different letters indicate significant differences (*p* < 0.05) according to Tukey’s test.

The results relating to hydrogen peroxide (H_2_O_2_) and malondialdehyde (MDA) levels, two key markers of oxidative stress, show significant variations depending on the treatments applied (Figure 5). The application of C and SWRT, whether used separately or in combination, led to a notable reduction in these compounds in argan seedlings, regardless of their age, compared to the controls. The C + SWRT combination proved to be the most effective, reducing H_2_O_2_ levels by 28% in 1Y seedlings and 27% in 2Y seedlings (Figure 5a). The separate application of C and SWRT also led to reductions, albeit more modest. The MDA concentrations followed similar trends to those of H_2_O_2_. The combined application of C + SWRT resulted in a significant decrease in MDA levels of 37% in 1Y seedlings and 23% in 2Y seedlings (Figure 5b). The effect of separate applications remains more pronounced in young seedlings: a 15% reduction with C and 31% with SWRT, compared to only 5 and 10%, respectively, in 2Y seedlings, compared to the controls.

#### 2.4.2. Total Soluble Sugars, Protein, and Proline Content

**Figure 6:**
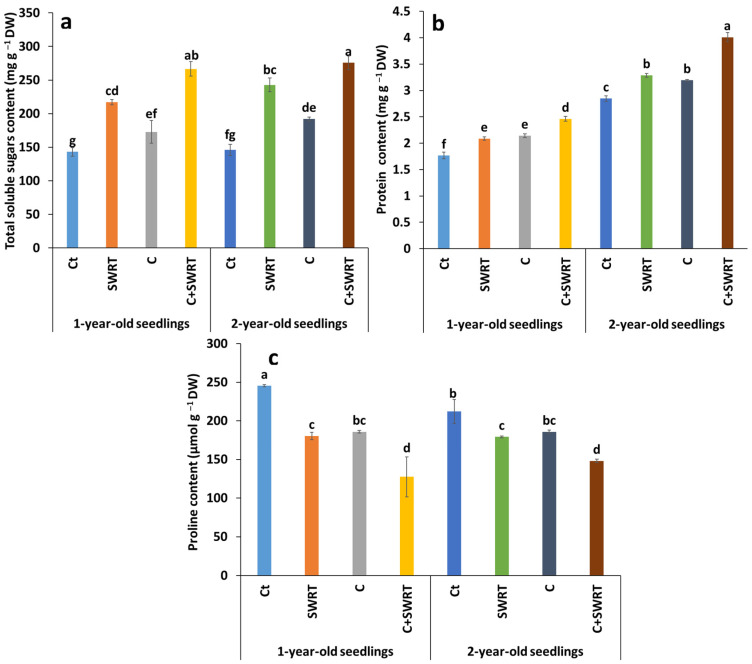
Effect of compost and/or SWRT application on total soluble sugars (**a**), protein (**b**) and proline (**c**) content in one- and two-year-old argan seedlings. Ct: control; SWRT: with SWRT; C: compost; C + SWRT: combined compost and SWRT. Different letters indicate significant differences (*p* < 0.05) according to Tukey’s test.

The results presented in Figure 6, clearly show the beneficial effect of the treatments on the accumulation of total soluble sugars, proteins, and proline in 1Y and 2Y argan seedlings. The combined application of compost and SWRT (C + SWRT) induced the most marked increases in soluble sugars, reaching 43% in 1Y seedlings and 51% in 2Y seedlings, compared to their respective controls. More moderate increases were also recorded with SWRT treatment alone, with 24% for 1Y seedlings and 29% for 2Y seedlings. Similar trends were observed for protein content. Separate application of compost led to a 19% increase in 1Y seedlings and a 3% increase in 2Y seedlings. When combined with SWRT technology, this treatment resulted in a greater improvement in protein content, reaching 26% for 1Y seedlings and 18% for 2Y seedlings. SWRT treatment alone also boosted protein content, with a 13% increase in younger seedlings and a 6% increase in older seedlings. In contrast, proline content followed an opposite trend. This parameter, which is naturally high in control seedlings, significantly decreased in all treatments applied alone or in combination. In 1Y seedlings, the most notable reductions were observed under SWRT alone and under C + SWRT, with decreases of approximately 40% compared to controls, while in 2Y seedlings, the decreases were more moderate: 20% under SWRT, 9% under C + SWRT, and only 5% under C alone.

#### 2.4.3. Polyphenol and Flavonoid Content

**Figure 7:**
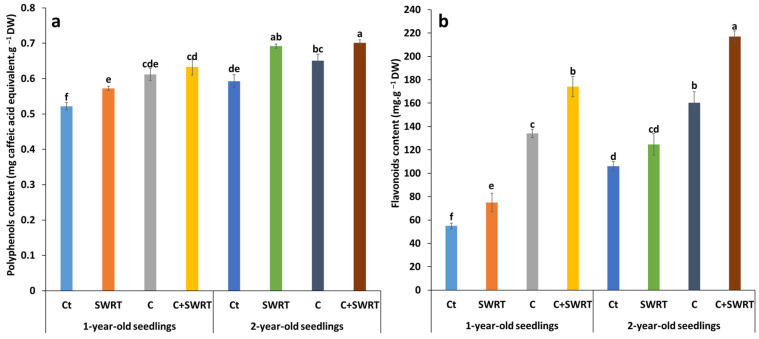
Effect of compost and/or SWRT application on polyphenols (**a**) and flavonoids (**b**) content in one- and two-year-old argan seedlings. Ct: control; SWRT: with SWRT; C: compost; C + SWRT: combined compost and SWRT. Different letters indicate significant differences (*p* < 0.05) according to Tukey’s test.

The results presented in Figure 7, highlight the effect of different treatments on polyphenols and flavonoids content in 1Y and 2Y argan seedlings. The combination of compost and SWRT technology led to a significant accumulation of polyphenols, with a 21% increase in 1Y seedlings and a 19% increase in 2Y seedlings, compared to their respective controls. The separate application of compost or SWRT also had a positive effect, although this varied depending on the age of the seedlings: in 1Y seedlings, the increases observed were 19% with compost and 10% with SWRT, while in 2Y seedlings, the enhancements were 19% for SWRT and 10% for compost. With regard to flavonoids content, the effects of the treatments were even more pronounced, regardless of the age of the seedlings. In 1Y seedlings, the combined C + SWRT treatment resulted in a dramatic 217% increase, followed by 144% with compost alone, while SWRT alone induced a more modest 38% increase. In 2Y seedlings, a similar trend was observed, with a 105% increase for the combined treatment, 51% for compost alone, and 18% for SWRT alone.

#### 2.4.4. Antioxidant Enzymes Activities

**Figure 8:**
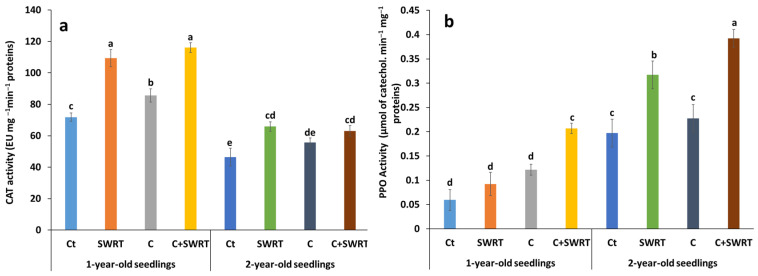
Effect of compost and/or SWRT application on catalase (CAT, (**a**)) and polyphenol oxidase (PPO, (**b**)) activity in one- and two-year-old argan seedlings. Ct: control; SWRT: with SWRT; C: compost; C + SWRT: combined compost and SWRT. Different letters indicate significant differences (*p* < 0.05) according to Tukey’s test.

The results presented in Figure 8 show a significant increase in the activity of catalase (CAT) and polyphenol oxidase (PPO) in 1Y and 2Y argan seedlings in response to the different treatments, compared to the controls. The combined application induced the highest marked increases for both enzymes, highlighting a synergistic effect on the antioxidant response. For CAT activity, 2Y seedlings showed a stronger response, with a 36% increase under C + SWRT, compared to 61% in 1Y seedlings. SWRT and C treatments alone induced smaller increases: 28 and 15%, respectively, in 2Y seedlings, compared to 30 and 7% in 1Y seedlings. Regarding PPO activity, the most pronounced effect was observed in younger seedlings, with a 167% increase under C + SWRT, followed by 87% under C alone, and 33% under SWRT. In 2Y seedlings, the increases were more moderate: 59% under C + SWRT, 18% under SWRT, and 1% under compost alone.

### 2.5. Principal Component Analysis

**Figure 9:**
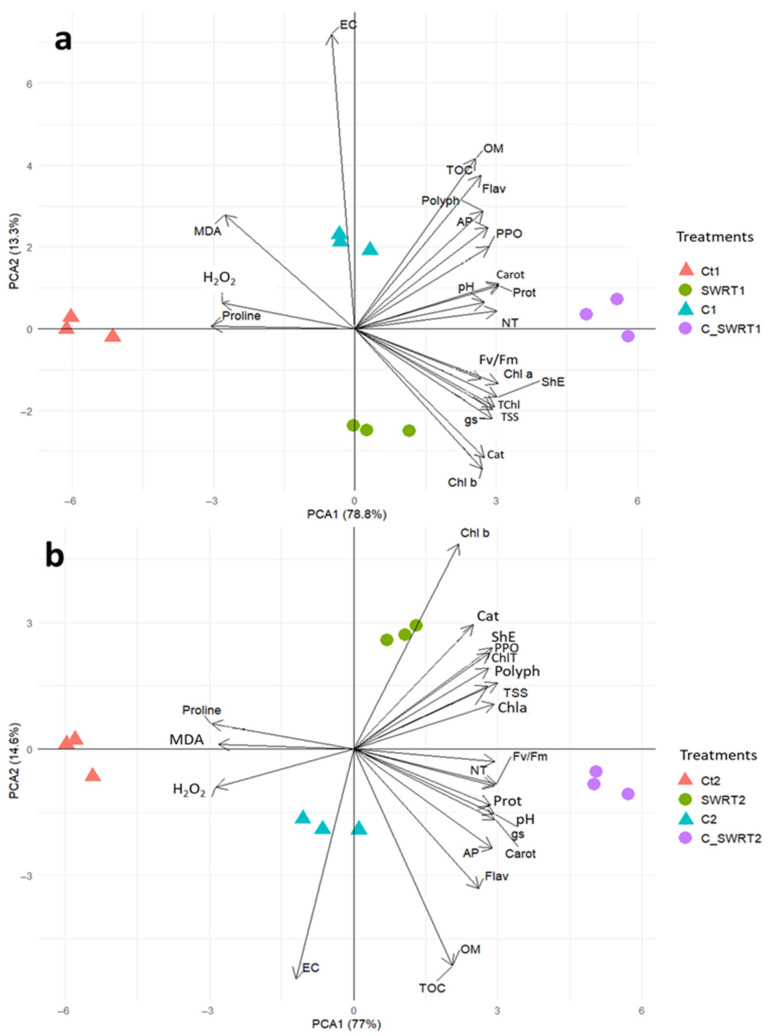
Principal component analysis Biplot (PCA) between treatments and evaluated parameters according to the 1-year-old seedlings (**a**) and 2-year-old seedlings (**b**). Ct: control; SWRT: subsurface water retention technology; C: compost; C_SWRT: compost + subsurface water retention technology. Treatments marked with (1) correspond to one-year-old seedlings, and those marked with (2) correspond to two-year-old seedlings. PPO: polyphenol oxidase activity; Prot: Protein content; Flav: Flavonoids content; Polyph: polyphenols content; Proline: Proline content; H_2_O_2_: hydrogen peroxide; MDA: malondialdehyde; EC: electrical conductivity; Cat: Catalase; Chl b: chlorophyll b; TChl: total chlorophyll; Fv/Fm: chlorophyll fluorescence; ShE: shoot elongation; NT: Total Nitrogen; Chl a: chlorophyll a; TSS: total soluble sugars content; gs: stomatal conductance; TOC: total organic carbon; OM: organic matter; AP: available phosphorus; Carot: carotenoids content.

The principal component analysis (PCA) carried out in this study for both 1Y and 2Y seedlings highlighted the relationships between the applied treatments and the measured parameters (Figure 9). The PCA for 1Y seedlings shows that 92.1% of the total variance is explained by the two components (PCA1: 78.8%; PC2: 13.3%) (Figure 9a). Similarly, for 2Y seedlings, these two principal components explain 91.6% of the total variance (PCA1: 77%; PC2: 14.6%) (Figure 9b). Correlation analysis reveals that Ct1, Ct2, C1 and C2 treatments are strongly negatively associated following PC1 axis with oxidative stress markers, including MDA, H_2_O_2_, proline, as well as EC. In contrast, SWRT1 and SWRT2 treatments are associated with physiological variables such as gs, Fv/Fm, Chl a, and Chl b. In addition, these treatments are correlated with soil parameters such as TOC, NT, and AP, as well as with some biochemical parameters including TSS, Cat, PPO, Flav, and Polyph. Finally, the majority of parameters are positively correlated with SWRT and C + SWRT treatments applied to 1Y or 2Y seedlings, while they show a strong negative correlation with Ct1 (Figure 9a) and Ct2 (Figure 9b).

### 2.6. Cluster Analysis and Dendrograms in a HeatMap Matrix

**Figure 10:**
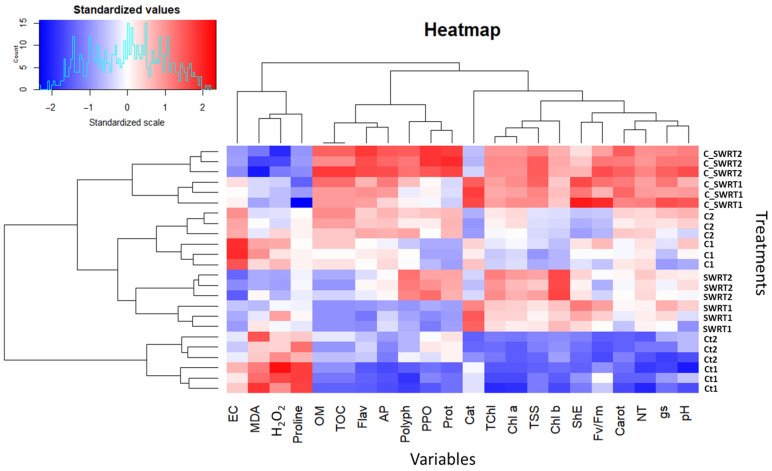
Heatmap illustrating the association between treatments and measured parameters in one-year-old seedlings and two-year-old seedlings, under different treatment conditions. The significance of the abbreviations is the same as in Figure 9.

The standardized heat map (Figure 10), enhanced with dendrograms, highlights the performance trends of the different sample groups. Blue indicates high values (better performance or high accumulation), while red indicates low values (poorer performance or low concentration). The results show that for both 1Y and 2Y seedlings, C_SWRT treatment groups (C_SWRT1 and C_SWRT2) stand out clearly: they show low levels of oxidative stress (red for MDA and H_2_O_2_) and high levels (blue) for most performance parameters, including chlorophylls (Chla, Chlb, TChl), proteins, carotenoids, and nutrients (NT, AP), as well as PPO enzyme activity. Conversely, the Ct groups (Ct1 and Ct2) often show the highest stress levels (red) and low values for these beneficial parameters, suggesting that C_SWRT treatment had a significant positive impact on plant health and physiology.

## 3. Discussion

Soil analysis findings demonstrated that the variation in soil physicochemical properties following the application of compost and SWRT technology aligns with established biogeochemical mechanisms, indicating a significant improvement in soil health and fertility for argan seedlings. The increase in soil pH towards a more alkaline state in compost-containing treatments is a well-documented effect. As it decomposes, compost releases base cations (Ca^2+^, Mg^2+^, K^+^) that neutralize soil acidity [21]. For its part, the SWRT technology alone does not have a major direct chemical impact on alkalinity, but its role in retaining water and nutrients [22] limits the leaching of base cations, thus indirectly helping to maintain pH stability [23]. As a result, the higher pH values observed when compost and SWRT are applied together can be explained by a functional synergy: SWRT technology maximizes the retention and concentration of alkalizing agents released by compost within the rhizosphere, enhancing and stabilizing the increase in pH and contributing significantly to improving soil health for argan seedlings. The physicochemical results of the soil show the synergistic effect of the combined treatment (C + SWRT) on improving nitrogen and phosphorus nutrition. This is explained by SWRT’s ability to maintain optimal and prolonged moisture in the root zone [15], thereby doubling the soil’s water retention capacity and creating ideal hydrobiological conditions for stimulating the activity of the soil microflora responsible for compost mineralization and, consequently, increasing the availability of these nutrients [24,25]. Finally, the increase in total organic carbon and organic matter (TOC and OM) in our results, especially with the application of the C + SWRT combination, is a direct consequence of the addition of compost, which is rich in organic matter [25,26]. In addition, the integration of SWRT creates synergy by maximizing the retention of this organic matter and nutrients in the root zone [15,27,28].

Similarly, shoot elongation findings revealed that the combined treatment (C + SWRT) resulted in a significantly higher increase in this parameter, while the treatment with compost alone showed the lowest level. One-year-old argan seedlings exhibited the highest levels of shoot elongation increase compared to two-year-old seedlings. Previous studies have demonstrated that SWRT significantly enhanced shoot elongation in both tomato [16] and argan [15] seedlings. Similarly, organic amendments such as compost have been shown to improve growth and shoot height of various plant species, including carob [12] and poplar [29].

Physiological traits constitute an important tool to study the effect of drought stress on many seedlings. The results of the present study revealed that the application of compost (C), SWRT, and their combination (C + SWRT) significantly promoted gs, Fv/Fm, and photosynthetic pigments content, compared to the controls, with higher plant fitness registered in C + SWRT treated-argan seedlings for both age groups. These improvements directly support plant physiological functions such as photosynthesis and resilience [30]. Previous study experiments further validate these findings, showing that SWRT significantly increases gs and Fv/Fm in tomato [16], cactus [28] and argan [15] seedlings. Furthermore, enriching the soil with organic compost, rich in carbon, phosphorus, and nitrogen, enhances the photosynthetic performance and metabolic efficiency of seedlings under drought stress [31]. Bouhadi et al. [32] also demonstrated that the desirable properties of compost, such as its high water-holding capacity and increased cation exchange capacity, are essential for improving soil moisture and nutrient accessibility. These improvements enable seedlings to develop a more robust photosynthetic apparatus, which is essential for energy production and growth in stressful environments. In addition, nutrients supplied by compost, such as nitrogen (N), are essential for the synthesis of chlorophyll and enzymes within chloroplasts, which are the sites of photosynthesis [33]. Previous research has demonstrated that the application of N-rich organic amendments significantly increases the Fv/Fm ratio in drought-stressed eggplants [34], carob [31], barley [32], and tomato [35]. On the other hand, this study’s findings showed that all treatments had positive physiological effects during the dry season, particularly in double-treated argan seedlings (C + SWRT) for both age groups. This suggests that these treatments improve soil water retention, which is critical for maintaining plant hydration and reducing drought stress [15,28,31,36]. Consistent with the physiological results, the application of compost, SWRT, and their combination significantly enhanced chlorophyll and carotenoid contents in *A. spinosa* seedlings across both age groups, with the most pronounced improvements observed in double-treated (C + SWRT) seedlings. These increases in pigment levels are closely associated with enhanced water retention and reduced oxidative stress, underscoring the role of compost and SWRT in supporting photosynthetic efficiency and drought resilience [15]. Carotenoids, in particular, function as specialized light-harvesting pigments that protect the photosynthetic apparatus by dissipating excess thermal energy under high light conditions, thereby contributing to the mitigation of oxidative stress [37]. Additionally, compost application further alleviates oxidative damage by increasing soil organic carbon and nutrient availability, which helps stabilize pigment synthesis under environmental stress [38].

Drought-induced reactive oxygen species (ROS) accumulation is triggered by chloroplast damage and disruption of mitochondrial electron transport chains, which can lead to membrane protein breakdown in seedlings via oxidative or proteolytic activity [39]. Under drought conditions, stress markers (MDA and H_2_O_2_) accumulation further disrupt cellular homeostasis, impairing critical physiological and metabolic processes [40]. In this study, the application of compost and SWRT either individually or in combination significantly reduced MDA and H_2_O_2_ levels in argan seedlings compared to untreated controls. The most pronounced reduction occurred in argan seedlings subjected to the combined treatment, underscoring the synergistic potential of these interventions. These results demonstrate the efficacy of compost and SWRT in mitigating oxidative damage under soil water scarcity conditions, corroborating earlier findings [15,16,39,41]. It has been reported that increased activity of antioxidant enzymes plays a crucial role in scavenging ROS and mitigating oxidative stress in plant cells under adverse conditions [42].

Compatible solutes such as sugars, proteins, and proline play a vital role in enabling seedlings to adapt to drought stress by contributing to cellular osmotic balance and stress tolerance mechanisms [43]. Our results revealed that the concentrations of soluble sugars and proteins were significantly higher in treated *A. spinosa* seedlings compared to the untreated controls across both age groups. The most notable increases were observed in seedlings receiving the combined treatment of compost and SWRT. The application of compost has been well-documented to enhance crop quality by increasing total soluble sugars and protein content, primarily through improved nutrient availability and uptake, particularly under stress conditions. Research has demonstrated protein content increases of up to 111% [44], alongside significant improvements in sugar accumulation [45] following compost application. By synergistically improving nutrient and water availability, the combined compost and SWRT treatment enhances osmolyte production. This leads to marked biochemical improvements that are vital for mature seedlings, ultimately boosting their stress resilience in arid environments [24]. On the other hand, the decline in proline content across all treatments suggests that the applied treatments reduced proline accumulation compared to the untreated control, with the effect being slightly more pronounced in younger seedlings. According to Akinmolayan and Adejumo [46], the application of compost has been shown to reduce the accumulation of proline in seedlings under stress conditions. For instance, in cowpea seedlings, compost reduced the accumulation of proline and glycine betaine under water stress conditions. This suggests that compost may alleviate stress, thereby reducing the need for proline accumulation. Similarly, improved water status due to SWRT might reduce the need for proline accumulation as a stress response [47].

The results of this study show that the application of SWRT alone or in combination with compost induced a significant increase in CAT and PPO activity in argan leaves, with a more pronounced increase under the C + SWRT treatment in both age groups. These results suggest an improvement in biochemical conditions and effective stimulation of plant antioxidant defense system. A study has shown that the application of SWRT is involved in the improvement of antioxidant enzymes (CAT, PPO, and peroxidase) under water stress conditions [16]. This mechanism of action can be explained by the ability of the SWRT membrane to retain water and essential nutrients in the rhizosphere, thereby reducing deep percolation [48]. This increased water availability helps maintain active cellular metabolism, promoting de novo biosynthesis of the enzyme proteins needed to detoxify ROS [49]. Furthermore, the synergistic effect observed with the addition of compost (C + SWRT) can be attributed to the supply of organic matter and nutrients (in the compost) that act as biostimulants, enhancing PPO and CAT activity to ensure better physiological tolerance and robust defense against environmental stressors [26,50,51].

Argan leaves are notably rich in phenolic compounds, including phenols and flavonoids [52]. These bioactive molecules play a crucial role in protecting seedlings against ROS. By acting as hydrogen or electron donors, phenolic compounds help neutralize singlet oxygen and scavenge free radicals, thereby shielding cellular proteins and lipid membranes from oxidative stress, particularly under drought conditions [16,53]. This study’s findings indicate that argan seedlings treated with compost alone or in combination with SWRT (C + SWRT) induced an increase in the synthesis of polyphenols and flavonoids, with a more pronounced increase in C + SWRT treatment in both age groups. This observation is consistent with previous research showing that compost amendments have positive effect on flavonoids levels in argan leaves [24] and significantly boost flavonoid levels in quinoa grain [54]. Moreover, total flavonoid content has been positively correlated with enhanced antioxidant activity against ROS in argan leaves [24]. Similarly, our findings align with evidence showing that compost improve the biochemical and antioxidant properties of *Moringa oleifera* leaves [55]. Taken together, these findings suggest that compost and C + SWRT treatments not only enhance the nutritional quality of *A. spinosa* but also strengthen its antioxidant defense system, thereby improving its capacity to withstand oxidative stress and environmental challenges [24,55].

Regarding the effect of age, as it was shown in PCA (Figure 9), our findings demonstrated that younger argan seedlings treated with C + SWRT exhibited the highest improvements in growth and pigments contents, indicating enhanced growth performance and pigment stability at early developmental stages. It has also been documented that seedling age at transplantation significantly affects growth rate and physiological performance across species. For instance, previous studies on *A. spinosa* and *Senegalia macrostachya* confirmed that juvenile seedlings achieve greater plant height and establishment success [19,56]. Younger seedlings generally display faster establishment, steeper growth curve and higher metabolic activity than older ones, leading to more efficient resource utilization and biomass accumulation [57]. The elevated protein, polyphenol, and flavonoid levels observed in younger C + SWRT-treated argan seedlings further suggest that this combination promotes both primary metabolism and the biosynthesis of antioxidant secondary metabolites critical for stress protection. According to Sarwar et al. [55], compost enhances nitrogen availability, promoting the synthesis of nitrogen-rich compounds such as protein, while phenolic compounds and flavonoids act as physical and chemical protectants against oxidative stress [58]. Furthermore, as it was shown in the Heatmap analysis (Figure 10) our results also indicate that younger seedlings treated with C + SWRT showed a greater significant increase in CAT and PPO levels. This age-dependent elevation of antioxidant and oxidative enzyme responses was associated with reduced lipid peroxidation and H_2_O_2_ accumulation, suggesting that the combined application of SWRT and compost effectively neutralizes ROS in argan seedlings. Consequently, this treatment reduces oxidative stress and membrane damage, particularly in younger seedlings, thereby improving their physiological resilience [59]. The marked increase in PPO activity observed in younger seedlings highlights its critical role in modulating ROS levels and protecting cellular components during stress conditions.

Moreover, the reduction in stress markers and oxidative stress due to proline accumulation in these seedlings supports the fact that C + SWRT promotes efficient ROS scavenging and osmotic adjustment, thereby enhancing tolerance to environmental stress [60]. Conversely, older argan seedlings treated with compost and SWRT exhibited significantly higher gs and Fv/Fm compared to younger seedlings, suggesting improved gas exchange capacity and reduced photoinhibition. These results suggest that, as seedlings mature, the development of hydraulic and anatomical structure improves water transport efficiency and coordination between stomatal regulation and photosynthetic activity, thereby supporting higher gs and overall photosynthetic performance [61]. The increased Fv/Fm ratio observed in older seedlings further reflects greater photochemical efficiency and a robust accumulation of photoprotective carotenoids, which facilitate the dissipation of excess light energy and mitigate photoinhibition. This observation is consistent with findings in *Mahonia oiwakensis*, where mature seedlings exhibited improved gas exchange and photoprotection under stress conditions [62]. Seo et al. [63] also found that older *Abies koreana* seedlings displayed higher photosynthetic capacity and were less sensitive to environmental stress compared to younger *A. koreana* individuals.

## 4. Materials and Methods

### 4.1. Plant Material

One- and two-year-old of *Argania spinosa* L. Skeels seedlings from the Essaouira ecotype were provided by the National Agency for the Development of Oasis Zones and Argan (ANDZOA), Essaouira, Morocco. The seedlings of the same age with homogeneous size (around 50 cm for one-year seedlings and 70 cm for two-year seedlings) were transplanted into the field following the experimental design described below.

### 4.2. Experimental Site and Design and Treatments

The field experiment was conducted in the Id Bouzid douar area (31°19′29.3′′ N, 9°32′32.8′′ W, 360 m altitude) within the Sidi Eljazouli commune, approximately 30 km southeast of Essaouira, Morocco, a region characterized by a semi-arid climate, with meteorological data provided in Figure S1. The 2.7-hectare experimental site was managed under a natural organic farming system without herbicides or chemical fertilizers. Weeds were manually removed as needed. Prior to transplantation, soil analysis showed the properties in Table 2.

The *Argania spinosa* L. Skeels seedlings were transplanted into the field, with the experimental design including four treatments: control seedlings (Ct) without any treatments, SWRT, compost (C), and a combination of compost and SWRT (C + SWRT). The treatments were randomly assigned with 25 replicates each per age group, for a total of 200 seedlings. The SWRT technology was applied by installing a square meter of biodegradable plastic (80 µm thick, 36-month lifespan) beneath each plant, buried at a depth of 60 cm in a U-shaped configuration to improve water and nutrient retention.

At the time of transplantation, 1.2 kg of compost per plant was added. Based on an apparent density of 0.6 kg/L, this amount is equivalent to 2 L of compost, incorporated into a total volume of 40 L per planting hole (5% *v*/*v*). To ensure substrate homogeneity, the compost was carefully mixed with the excavated soil before planting the seedlings. The green waste-based compost used in this experiment was produced in the composting unit of the Faculty of Science, Semlalia, Marrakech, Morocco. The raw material consisted of 100% dead leaves from deciduous trees collected from the botanical garden of the faculty. The composting process was carried out in accordance with the protocol described by Meddich et al. [64]. In short, the raw material was shredded and arranged in aerobic windrows. The piles were turned manually every 15 days to ensure adequate aeration and homogenization. The moisture content was maintained at approximately 60–70% of water retention capacity through regular watering. The thermophilic phase was monitored, and the composting process was considered complete after approximately 6 months, once the temperature stabilized at ambient levels and the C/N ratio reached maturity standards. The physicochemical characteristics of the compost are shown in Table 3. The field experiment lasted from March 2023 to February 2024, and the seedlings were monthly irrigated with 50 L of water per plant by filling the microbasins surrounding each seedling.

### 4.3. Soil Properties Measurements

Soil sampling was carried out in two stages: (i) prior to the experiment, a composite sample was taken at random from across the site at a depth of 20–30 cm in order to determine the initial physicochemical properties (Table 2); (ii) after the experiment, at the end of the trial, rhizosphere soil samples were collected at the same depth (20–30 cm) for each treatment and stored in sampling bags for textural and physicochemical analysis.

Soil pH and EC were measured by mixing 5 g of 2 mm-sieved soil with 25 mL of distilled water (1:5 *v*/*v* ratio), shaking for 30 min, and then taking measurements using a pH-meter and conductivity meter (pH 1970i and Cond 1970i, WTW GmbH, Weilheim, Germany). The TOC was determined following the Aubert method [65] and the organic matter (OM) content was subsequently calculated by multiplying the TOC percentage by the Van Bemmelen factor of 1.724, while AP was assessed spectrophotometrically at 820 nm based on the reduction of a phosphoric acid-molybdate complex according to Olsen and Sommers [66]. The total nitrogen (TN) content was analysed using the Kjeldahl method as described by Bremner [67] using an automated distiller (KJA-9840 Model, Weifang, China).

### 4.4. Growth and Physiological Parameters Measurements

The shoot elongation of argan seedlings was assessed by measuring their total height (H), defined as the distance from the collar to the tip of the apical meristem. These measurements were taken monthly using a measuring tape. The monthly elongation rate (%E) was then calculated to quantify relative growth over the study period. This quantification is based on the formula: $\%E=\frac{Lf-Li}{Li}\;100$, where Li represents the initial height of the plant at the beginning of the month and Lf its final height at the beginning of the following month, thus expressing the increase in height as a percentage of the initial size.

Physiological parameters were assessed by measuring the maximal photochemical efficiency of PSII (Fv/Fm) and stomatal conductance (gs) in fully expanded and mature argan leaves. The Fv/Fm ratio was determined using a fluorometer (Opti-Sciences OSI 30p, Hudson, NY, USA) after acclimating leaves to darkness for 30 min using clips. The efficiency of PSII was quantified as Fv/Fm, where Fv represents variable fluorescence calculated as Fv = Fm − F0, Fm is maximum fluorescence, and F0 is initial fluorescence. The gs was measured using a portable porometer (Leaf Porometer LP1989, Decagon Device, Inc., Washington, DC, USA) on the abaxial leaf surface during the morning hours (9:00 a.m. to 11:00 a.m.) of sunny days. Five measurements per treatment were conducted on leaves from the same row in the upper part of the plant.

The concentrations of chlorophyll a, chlorophyll b, total chlorophyll, and carotenoids were determined following the method outlined by Arnon [68]. These photosynthetic pigments were extracted from frozen leaf samples using 80% (*v*/*v*) acetone. The extract was centrifuged at 10,000× *g* for 10 min at 4 °C, and the supernatant was collected. The absorbance (A) of the supernatant was measured using a Spectrophotometer (UV-3100PC Spectrophotometer, VWR International, Radnor, PA, USA) at 663, 645, and 480 nm to quantify chlorophyll a (Chl a), chlorophyll b (Chl b), and carotenoids (Car), respectively.

### 4.5. Biochemical Leaf Parameters Measurements

#### 4.5.1. Malondialdehyde and Hydrogen Peroxide Content Quantification

Malondialdehyde (MDA) content was determined by spectrophotometry at 760 nm according to Savicka and Škute [69]. The extract was prepared by mixing 0.25 g of the sample with trichloroacetic acid at 10% (TCA). The mixture was then centrifuged at 18,000× *g* for 20 min and 1 mL of the supernatant was mixed with 2.5 mL of thiobarbituric acid (TBA) at 0.6%. After incubation at 95 °C for 30 min, the tubes were placed in an ice bath. The resulting chromogen was measured at 450, 532, and 600 nm. MDA content was calculated using the following equation:

[MDA] = 6.45 × (A_532_ − A_600_) − 0.565 × A_450_

A = Absorbance

Hydrogen peroxide (H_2_O_2_) content was assessed according to Aebi [70]. An amount of 0.25 g of frozen crushed leaves were mixed with 5 mL of TCA 10% (*w*/*v*) and then centrifuged at 15,000× *g* for 15 min at 4 °C. The reaction mixture included 2 mL of the extract, 1 mL of potassium iodide (1 M), and 0.5 mL of potassium phosphate buffer (10 mM, pH 7). The absorbance was measured at 390 nm after one hour of incubation in the dark. Concentrations of H_2_O_2_ were determined using a standard H_2_O_2_ curve.

#### 4.5.2. Total Soluble Sugar and Proline Content Quantification

The total soluble sugar (TSS) content was determined using 0.1 g of frozen samples homogenized in 4 mL of 80% (*v*/*v*) ethanol. The resulting supernatant was mixed with 0.25 mL of 5% (*v*/*w*) phenol and 1.25 mL of concentrated sulfuric acid. Absorbance was measured at 485 nm using a UV-3100PC spectrophotometer, following the method of DuBois et al. [71]. Proline content was quantified following the method of Carillo et al. [72]. Briefly, 0.1 g of fresh material was ground in 4 mL of 40% ethanol (*v*/*v*). The extract was stored at 4 °C overnight. The resulting ethanol extract was then treated with 1 mL of a mixture of 60% acetic acid, 1% ninhydrin, and 20% ethanol. The reaction mixture was kept at 90 °C for 20 min. The absorbance was read at 520 nm.

#### 4.5.3. Proteins Content and Antioxidant Enzymes Activity Measurements

An amount of 0.1 g of frozen leaf samples was homogenized in a cold mortar with 4 mL of 1 M phosphate buffer (pH 7) containing 5% polyvinylpolypyrrolidone and 0.1 mM ethylenediaminetetraacetic acid. The homogenate was centrifuged at 18,000× *g* for 15 min at 4 °C. The supernatant was then used to measure protein content and antioxidant enzyme activities. The total content of soluble protein in the leaf samples was determined according to the method of Bradford [73]. The absorbance was read at 595 nm, and bovine serum albumin was used as a protein standard. Catalase (CAT) activity was measured according to Aebi [70] by monitoring the decrease in absorbance at 240 nm for 3 min after consumption of H_2_O_2_ substrate at 240 nm for 3 min. Polyphenoloxidase (PPO) activity was assessed by monitoring catechol oxidation at 410 nm according to Gauillard et al. [74]. The reaction mixture used contained K_2_HPO_4_/KH_2_PO_4_ buffer (100 mM, pH 6), catechol (50 mM), and the enzyme extract. The CAT, and PPO activities were expressed in unit mg protein^−1^.

Total phenolic content (TPC) was measured in leaf sample extracts using the Folin–Ciocalteu method of Singleton and Rossi [75] with slight modifications. Briefly, 2.5 mL of 1 N Folin–Ciocalteu reagent solution was added to an aliquot of 250 µL of the extract solution. After 3 min of incubation at room temperature, 250 µL of 10% sodium carbonate solution was added and kept in the dark for 90 min. The absorbance was then read spectrophotometrically at 760 nm.

The total flavonoid content was determined in accordance with the method of Al-Farsi and Lee [76]. Briefly, 60 μL of 5% NaNO_2_ and 60 μL of AlCl_3_ (10%) were added to the methanol extracts (200 μL). Subsequently, 400 μL of 1 M NaOH was added, and the absorbance was read at 510 nm with quercetin as the standard. Results were expressed as mg quercetin equivalents per 100 mg DW.

### 4.6. Statistical Data Analysis

Data presented are averages ± standard errors. Values are based on five replicates for growth and physiological parameters and three replicates for biochemical analyses and soil physico-chemical traits. Statistical analysis was performed using the statistical software R v4.1.2 (R Core Team, 2021) [77] on RStudio (V 4.04.0 + 735; RStudio Team, 2024) for Windows, using analysis of variance (ANOVA) to test the effects of treatment and age on leaf physiological and biochemical parameters and on soil properties. Statistical significance was determined at a threshold of *p* < 0.05, which means that differences in the mean values were considered to be significant if the probability of error was less than 5%.

## 5. Conclusions

The present study demonstrated that the integration of compost and SWRT provides a synergistic cultivation environment for *A. spinosa* establishment that significantly outperforms individual treatments. By simultaneously optimizing soil fertility and water availability, this dual strategy provides a robust physiological buffer against the oxidative stress inherent to arid ecosystems. Furthermore, our findings establish that seedling age at transplanting significantly influenced plant performance where younger seedlings exhibit superior growth performance, whereas older seedlings provide enhanced physiological and enzymatic resilience under extreme conditions. Ultimately, transitioning from traditional planting methods to this technologically enhanced, age-specific strategy provides a scalable and sustainable blueprint for restoring degraded Argan forests in the face of escalating desertification

## Figures and Tables

**Table 1:** Effect of compost and/or SWRT application on soil physicochemical parameters before and after the experiment.

	Before	1-Year-Old Seedlings				2-Year-Old Seedlings			
		Ct	SWRT	C	C + SWRT	Ct	SWRT	C	C + SWRT
pH	7.40 ± 0.03 ^e^	7.90 ± 0.06 ^d^	7.99 ± 0.06 ^bcd^	8.01 ± 0.06 ^a–d^	8.10 ± 0.03 ^ab^	7.93 ± 0.40 ^cd^	8.03 ± 0.01 ^abc^	8.03 ± 0.04 ^abc^	8.13 ± 0.02 ^a^
EC (mS.cm^−1^)	282.12 ± 11.74 ^a^	237.33 ± 6.03 ^b^	205.66 ± 5.03 ^de^	272.33 ± 5.51 ^a^	229.00 ± 3.61 ^bc^	217.66 ± 4.93 ^cd^	191.33 ± 5.85 ^e^	247.60 ± 3.05 ^b^	200.60 ± 2.51 ^de^
AP (ppm)	38.40 ± 0.06 ^fg^	38.40 ± 0.38 ^g^	49.90 ± 2.86 ^e^	57.12 ± 2.62 ^cd^	64.09 ± 4.23 ^b^	44.11 ± 1.40 ^f^	56.11 ± 1.13 ^d^	62.08 ± 0.76 ^bc^	70.98 ± 1.13 ^a^
NT (g. Kg^−1^)	2.08 ± 0.29 ^d^	3.06 ± 0.32 ^c^	5.17 ± 0.03 ^b^	5.30 ± 0.18 ^b^	6.12 ± 0.18 ^a^	3.40 ± 0.21 ^c^	5.45 ± 0.09 ^b^	5.43 ± 0.06 ^b^	6.18 ± 0.09 ^a^
TOC (%)	0.48 ± 0.02 ^c^	0.39 ± 0.03 ^c^	0.42 ± 0.00 ^c^	0.59 ± 0.03 ^b^	0.69 ± 0.03 ^a^	0.48 ± 0.05 ^c^	0.46 ± 04 ^c^	0.66 ± 0.04 ^ab^	0.74 ± 0.05^a^
OM (%)	0.88 ± 0.04 ^c^	0.67 ± 0.05 ^c^	0.72 ± 0.00 ^c^	1.01 ± 0.05 ^b^	1.18 ± 0.06 ^a^	0.82 ± 0.08 ^c^	0.79 ± 0.06 ^c^	1.13 ± 0.06 ^ab^	1.27 ± 0.08 ^a^

Ct: control; SWRT: subsurface water retention technology; C: compost; C + SWRT: compost + subsurface water retention technology; EC: electrical conductivity; AP: available phosphorus; NT: total nitrogen; TOC: total organic carbon; OM: organic matter. Values represent the mean ± standard error (SE) of three replicates (*n* = 3). Different letters within the same row indicate statistically significant differences at *p* < 0.05 according to Tukey’s test.

**Table 2:** Soil characteristics prior to transplantation.

Parameter	Sand (%)	Silt (%)	Clay (%)	AP (ppm)	NT (g. Kg^−1^)	TOC (%)	OM (%)	pH	EC (µS/cm)	WRB Classification
Value	34.58	48.51	16.84	38.40	2.08	0.48	0.88	7.40	282.12	Eutric Siltic Cambisol

AP, available phosphorus; NT, total nitrogen; TOC, total organic carbon; OM, Organic Matter; EC, electrical conductivity; WRB, world reference base for soil resources.

**Table 3:** Physicochemical characterization of green waste compost.

Elements	C	N	C/N	O	Fe	Na	Mg	Si	P	S	Cl	K	Ca
Weight (%)	20.70	1.07	19.30	44.60	0.70	0.20	3.00	14.10	0.30	0.40	0.70	2.80	9.00

C, Carbon; N, Nitrogen, C/N, Carbon to Nitrogen ratio; O, Oxygen; Fe, Iron, Na, Sodium; Mg, Magnesium; Si, Silicon; P, Phosphorus; S, Sulfur; Cl, Chlorine; K, Potassium; Ca, Calcium.

## Data Availability

Data are contained within the article and Supplementary Materials.

## Supplementary Materials

The following supporting information can be downloaded at: https://www.mdpi.com/article/10.3390/plants15030365/s1, Figure S1: Meteorological data during the experiment period.

## References

[1] Toscano S., Farieri E., Ferrante A., Romano D. (2016). Physiological and Biochemical Responses in Two Ornamental Shrubs to Drought Stress. Front. Plant Sci..

[2] Díaz-barradas M.C., Zunzunegui M. (2010). Seasonal Physiological Responses of *Argania spinosa* Tree from Mediterranean to Semi-Arid Climate. Plant Soil.

[3] El Ghazali H., Harrouni C., Daoud S., Tlemçani N.B. (2021). Impact of Climate Change on the Argan Biosphere Reserve (ABR) in Morocco. Preprints.

[4] Lamhamedi M.S., Pepin S., Khasa D. (2023). The Production Chain of Tree Seedlings, from Seeds to Sustainable Plantations: An Essential Link for the Success of Reforestation and Restoration Programs in the Context of Climate Change. Forests.

[5] Moukrim S., Lahssini S., Rhazi M., Alaoui H.M., Benabou A., Wahby I., El Madihi M., Arahou M., Rhazi L. (2019). Climate Change Impacts on Potential Distribution of Multipurpose Agro-Forestry Species: *Argania spinosa* (L.) Skeels as Case Study. Agrofor. Syst..

[6] Nait Douch A., Boukhalef L., El Asbahani A., Al-namazi A.A., ElMehrach K., Bouqbis L., Touaf M., Ain-Lhout F. (2022). Photosynthetic Behaviorof *Argania spinosa* (L.) Skeels Induced under Grazed and Ungrazed Conditions. Sustainability.

[7] Fassih B., Ait-El-Mokhtar M., Douch A.N., Boutasknit A., Ben-Laouane R., Aganchich B., Wahbi S. (2025). The Impact of Browsing Intensity on Argan Trees in the Essaouira Region of Morocco. J. Arid. Environ..

[8] Defaa C., Elantry S., El Alami S.L., Achour A., El Mousadik A., Msanda F. (2015). Effects of Tree Shelters on the Survival and Growth of *Argania spinosa* Seedlings in Mediterranean Arid Environment. Int. J. Ecol..

[9] Timzioura R., Ezzine S., Benomar L., Lamhamedi M.S., Ettaqy A., Zine A., Abidine E., Zaher H., Khasa D.P., Pepin S. (2025). Bibliometric Analysis of Argan (*Argania spinosa* (L.) Skeels) Research: Scientific Trends and Strategic Directions for Climate-Resilient Ecosystem Management. Forests.

[10] Fitzpatrick G. (2001). Compost Utilization in Ornamental and Nursery Crop Production Systems. Compost Utilization in Horticultural Cropping Systems.

[11] Duong T.T.T., Penfold C., Marschner P. (2012). Amending Soils of Different Texture with Six Compost Types: Impact on Soil Nutrient Availability, Plant Growth and Nutrient Uptake. Plant Soil.

[12] Boutasknit A., Baslam M., Ait-El-Mokhtar M., Anli M., Ben-Laouane R., Ait-Rahou Y., Mitsui T., Douira A., El Modafar C., Wahbi S. (2021). Assemblage of Indigenous Arbuscular Mycorrhizal Fungi and Green Waste Compost Enhance Drought Stress Tolerance in Carob (*Ceratonia siliqua* L.) Trees. Sci. Rep..

[13] Close D.C., Paterson S., Corkrey R., McArthur C. (2010). Influences of Seedling Size, Container Type and Mammal Browsing on the Establishment of Eucalyptus Globulus in Plantation Forestry. New For..

[14] Aoda M.I., Smucker A.J.M., Majeed S.S., Mohammed H.A., Al-Sahaf F.H., Robertson G.P. (2021). Novel Root Zone Soil Water Retention Improves Production with Half the Water in Arid Sands. Agron. J..

[15] Fassih B., Ait-el-mokhtar M., Douch A.N., Boutasknit A., Ben-Laouane 4 R., Aganchich B., Wahbi S. (2024). Combined Effect of Subsurface Water Retention Technology and Arbuscular Mycorrhizal Fungi on Growth, Physiology and Biochemistry of Argan Seedlings under Field Conditions. Plants.

[16] Lahbouki S., Meddich A., Ben-Laouane R., Outzourhit A., Pari L. (2022). Subsurface Water Retention Technology Promotes Drought Stress Tolerance in Field-Grown Tomato. Energies.

[17] Hartmann M., Six J. (2023). Soil Structure and Microbiome Functions in Agroecosystems. Nat. Rev. Earth Environ..

[18] Ren Y., Zhu J., Hussain N., Ma S., Ye G., Zhang D., Hua S. (2014). L’â Ge et La Qualityé Des Plantules Au Repiquage Affectent Le Rendement Grainier Du Canola (*Brassica napus* L.). Can. J. Plant Sci..

[19] Oumahmoud M., Alouani M., Elame F., Tahiri A., Bouharroud R., Qessaoui R., Wifaya A., Amesmoud G., Koufan M. (2024). Nursery Production, Acclimatization, and Orchard Transplantation of *Argania spinosa*: Evaluating the Impact of Costs and Plant Age. Sci. Hortic..

[20] Azad S.F.D., Ali M.Y., Quddus K.G., Sarker B.C., Ray J. (2022). Effect of Seedling Age on Growth and Yield of Local Aman Rice Varieties in the Low Land Area of Southwestern Coastal Bangladesh. Int. J. Plant Soil Sci..

[21] Adugna G. (2016). A Review on Impact of Compost on Soil Properties, Water Use and Crop Productivity. Acad. Res. J. Agric. Sci. Res..

[22] Kubuga C.K. (2025). Dialogues in Health Alternative Community-Based Gardening and Water Banks for Micronutrients Intake among Women in Northern Ghana. Dialogues Health.

[23] Guber A.K., Smucker A.J.M., Berhanu S., Miller J.M.L. (2015). Subsurface Water Retention Technology Improves Root Zone Water Storage for Corn Production on Coarse-Textured Soils. Vadose Zone J..

[24] Chabbi N., Labbassi S., Afi C., Chafiki S., Telmoudi M., Tiouidji F.E., Wifaya A., Bouharroud R., Tahiri A., Qessaoui R. (2024). Mineral and Organic Fertilizers’ Effect on the Growth of Young Argane Trees (*Argania spinosa* L.) and Soil Properties under Vulnerable Conditions. Plants.

[25] Chakhchar A., Lamaoui M., El Kharrassi Y., Bourhim T., Filali-Maltouf A., El Modafar C. (2020). A Review on the Root System of *Argania spinosa*. Curr. Agric. Res. J..

[26] El Moussaoui H., Bouqbis L. (2022). Interactive Effect of Biochar and Bio-Compost on Starting Growth and Physiologic Parameters of Argan. Sustainability.

[27] Al-shami Y.A.O., Al-timimi M.I.A. (2015). The Effect of Using Subsurface Water Retention Technology on the Available Content of Soil Nutrients under Subsurface Drip Irrigation System. Misan J. Agric. Environ. Sci..

[28] Lahbouki S., Ech-Chatir L., Er-Raki S., Outzourhit A., Meddich A. (2022). Improving Drought Tolerance of Opuntia Ficus-Indica under Field Using Subsurface Water Retention Technology: Changes in Physiological and Biochemical Parameters. Can. J. Soil Sci..

[29] Simiele M., De Zio E., Montagnoli A., Terzaghi M., Chiatante D., Scippa G.S., Trupiano D. (2022). Biochar and/or Compost to Enhance Nursery-Produced Seedling Performance: A Potential Tool for Forest Restoration Programs. Forests.

[30] Baslam M., Mitsui T., Hodges M., Priesack E., Herritt M.T., Aranjuelo I., Sanz-Sáez Á. (2020). Photosynthesis in a Changing Global Climate: Scaling Up and Scaling Down in Crops. Front. Plant Sci..

[31] Boutasknit A., Ait-El-Mokhtar M., Fassih B., Ben-Laouane R., Wahbi S., Meddich A. (2024). Effect of Arbuscular Mycorrhizal Fungi and Rock Phosphate on Growth, Physiology, and Biochemistry of Carob under Water Stress and after Rehydration in Vermicompost-Amended Soil. Metabolites.

[32] Bouhadi M., Javed Q., Kovačević T.K., Ban D., Heath D., Černe M. (2025). Enhancing Drought Tolerance in Barley (*Hordeum vulgare* L.) Through the Application of Olive Pomace Compost. Appl. Sci..

[33] Chen L., Xu M., Cheng Z., Yang L. (2024). Effects of Nitrogen Deficiency on the Photosynthesis, Chlorophyll a Fluorescence, Antioxidant System, and Sulfur Compounds in Oryza Sativa. Int. J. Mol. Sci..

[34] Abd El-Mageed T.A., Abdelkhalik A., Abd El-Mageed S.A., Semida W.M. (2021). Co-Composted Poultry Litter Biochar Enhanced Soil Quality and Eggplant Productivity Under Different Irrigation Regimes. J. Soil Sci. Plant Nutr..

[35] El Amerany F., Rhazi M., Wahbi S., Taourirte M., Meddich A. (2020). Scientia Horticulturae The effect of Chitosan, Arbuscular Mycorrhizal Fungi, and Compost Applied Individually or in Combination on Growth, Nutrient Uptake, and Stem Anatomy of Tomato. Sci. Hortic..

[36] Zgallai H., Zoghlami R.I., Annabi M., Zarrouk O., Jellali S., Hamdi H. (2024). Mitigating Soil Water Deficit Using Organic Waste Compost and Commercial Water Retainer: A Comparative Study under Semiarid Conditions. Euro-Mediterr. J. Environ. Integr..

[37] Rao M.J., Duan M., Zhou C., Jiao J., Cheng P., Yang L., Wei W., Shen Q., Ji P., Yang Y. (2025). Antioxidant Defense System in Plants: Reactive Oxygen Species Production, Signaling, and Scavenging During Abiotic Stress-Induced Oxidative Damage. Horticulturae.

[38] Oueld Lhaj M., Moussadek R., Zouahri A., Sanad H., Saafadi L., Mdarhri Alaoui M., Mouhir L. (2024). Sustainable Agriculture Through Agricultural Waste Management: A Comprehensive Review of Composting’s Impact on Soil Health in Moroccan Agricultural Ecosystems. Agriculture.

[39] Soussani F.E., Boutasknit A., Ben-Laouane R., Benkirane R., Baslam M., Meddich A. (2023). Arbuscular Mycorrhizal Fungi and Compost-Based Biostimulants Enhance Fitness, Physiological Responses, Yield, and Quality Traits of Drought-Stressed Tomato Plants. Plants.

[40] Hasanuzzaman M., Bhuyan M.H.M.B., Parvin K., Bhuiyan T.F., Anee T.I., Nahar K., Hossen M.S., Zulfiqar F., Alam M.M., Fujita M. (2020). Regulation of Ros Metabolism in Plants under Environmental Stress: A Review of Recent Experimental Evidence. Int. J. Mol. Sci..

[41] Anli M., Boutasknit A., Ait-El-Mokhtar M., Ben-Laouane R., Ait-Rahou Y., Fakhech A., Meddich A. (2022). Improving Lettuce Yield and Quality of an Agricultural Soil Using a Combination of Arbuscular Mycorrhizal Fungus and Phosphate-Green Wastes Compost. Gesunde Pflanz..

[42] Dumanović J., Nepovimova E., Natić M., Kuča K., Jaćević V. (2021). The Significance of Reactive Oxygen Species and Antioxidant Defense System in Plants: A Concise Overview. Front. Plant Sci..

[43] Haghpanah M., Hashemipetroudi S., Arzani A., Araniti F. (2024). Drought Tolerance in Plants: Physiological and Molecular Responses. Plants.

[44] Ouhaddou R., Ben-Laouane R., Lahlali R., Anli M., Ikan C., Boutasknit A., Slimani A., Oufdou K., Baslam M., Ait Barka E. (2022). Application of Indigenous Rhizospheric Microorganisms and Local Compost as Enhancers of Lettuce Growth, Development, and Salt Stress Tolerance. Microorganisms.

[45] Maganizo Kamanga R., Matuntha I., Chawanda G., Mtaya J., Chasweka T., Dzimbiri C., Stevens J., Nyasulu M., Chiwasa H., Sefasi A. (2024). Exploration of Agronomic Efficacy and Drought Amelioration Ability of Municipal Solid Waste-Derived Co-Compost on Lettuce and Maize. Sustainability.

[46] Akinmolayan T.V., Adejumo S.A. (2022). Pre-Sowing Seed Treatment with Proline, Glycine Betaine, and Soil Amendment with Compost as Strategies for Improving Yield and Drought Tolerance in Cowpea. J. Soil Sci. Plant Nutr..

[47] Li B., Ni J., Wang J., Xiong Z., Wang J., Zhu L., Liu S. (2012). Effect of Water-Retaining Agent on Growth and Development of Three Local Legumes on Lead-Zinc Tailings of Lanping. Adv. Mater. Res..

[48] Nkurunziza L., Chirinda N., Lana M., Sommer R., Karanja S., Rao I., Romero Sanchez M.A., Quintero M., Kuyah S., Lewu F. (2019). The Potential Benefits and Trade-Offs of Using Sub-Surface Water Retention Technology on Coarse-Textured Soils: Impacts of Water and Nutrient Saving on Maize Production and Soil Carbon Sequestration. Front. Sustain. Food Syst..

[49] Rajput V.D., Harish, Singh R.K., Verma K.K., Sharma L., Quiroz-Figueroa F.R., Meena M., Gour V.S., Minkina T., Sushkova S. (2021). Recent Developments in Enzymatic Antioxidant Defence Mechanism in Plants with Special Reference to Abiotic Stress. Biology.

[50] Boutasknit A., Baslam M., Anli M., Ait-El-Mokhtar M., Ben-Laouane R., Ait-Rahou Y., El Modafar C., Douira A., Wahbi S., Meddich A. (2022). Impact of Arbuscular Mycorrhizal Fungi and Compost on the Growth, Water Status, and Photosynthesis of Carob (*Ceratonia siliqua*) under Drought Stress and Recovery. Plant Biosyst..

[51] Ait Rahou Y., Ait-El-Mokhtar M., Anli M., Boutasknit A., Ben-Laouane R., Douira A., Benkirane R., El Modafar C., Meddich A. (2021). Use of Mycorrhizal Fungi and Compost for Improving the Growth and Yield of Tomato and Its Resistance to Verticillium Dahliae. Arch. Phytopathol. Plant Prot..

[52] Hilali M., El Monfalouti H., Kartah B.E. (2020). Study of the Flavonoids and Secondary Metabolites of the Argan Tree (*Argania spinose* L.). Online J. Anim. Feed Res..

[53] Kalogianni A.I., Lazou T., Bossis I., Gelasakis A.I. (2020). Natural Phenolic Compounds for the Control of Oxidation, Bacterial Spoilage, and Foodborne Pathogens in Meat. Foods.

[54] Benaffari W., Soussani F., Boutasknit A., Toubali S. (2024). Arbuscular Mycorrhizal Fungi Improve Drought Tolerance of Quinoa Grown in Compost-Amended Soils by Altering Primary and Secondary Metabolite Levels. Phyton.

[55] Sarwar M., Patra J.K., Ali A., Maqbool M., Arshad M.I. (2020). Effect of Compost and NPK Fertilizer on Improving Biochemical and Antioxidant Properties of Moringa Oleifera. S. Afr. J. Bot..

[56] Zida D., Sanou L., Savadogo P., Jonas K., Sawadogo L. (2023). Trees, Forests and People Transplanted Seedling Age and Watering Effects on the Field Performance of *Senegalia macrostachya* (Rchb. Ex DC.) Kyal. & Boatwr., a High-Valued Indigenous Fruit Tree Species in Burkina Faso. Trees For. People.

[57] Xu C., Kim S.H., Kim J.K., Heo J.Y., Vu N.T., Choi K.Y., Kim I.S., Jang D.C. (2021). The Effect of Transplant Age on Vegetable Growth Characteristic in a Cylindrical Paper Pot System. Hortic. Environ. Biotechnol..

[58] Rankenberg T., Geldhof B., van Veen H., Holsteens K., Van de Poel B., Sasidharan R. (2021). Age-Dependent Abiotic Stress Resilience in Plants. Trends Plant Sci..

[59] Mekkaoui F., Ait-El-Mokhtar M., Zaari Jabri N., Amghar I., Essadssi S., Hmyene A. (2024). The Use of Compost and Arbuscular Mycorrhizal Fungi and Their Combination to Improve Tomato Tolerance to Salt Stress. Plants.

[60] Zhang S. (2023). Recent Advances of Polyphenol Oxidases in Plants. Molecules.

[61] Beikircher B., Sack L., Ganthaler A., Losso A., Mayr S. (2021). Hydraulic-stomatal Coordination in Tree Seedlings: Tight Correlation across Environments and Ontogeny in Acer Pseudoplatanus. New Phytol..

[62] Chen C.I., Lin K.H., Lin T.C., Huang M.Y., Chen Y.C., Huang C.C., Wang C.W. (2023). Responses of Photosynthesis and Chlorophyll Fluorescence during Light Induction in Different Seedling Ages of Mahonia Oiwakensis. Bot. Stud..

[63] Seo H.-N., Chae S.-B., Lim H.-I., Han S.-H., Lee K. (2021). Selecting Appropriate Seedling Age for Restoration Using Comparative Analysis of Physiological Characteristics by Age in Abies Koreana Wilson. J. For. Environ. Sci..

[64] Meddich A., Oufdou K., Boutasknit A., Raklami A., Tahiri A., Ben-Laouane R., Ait-El-Mokhtar M., Anli M., Mitsui T., Wahbi S. (2019). Use of Organic and Biological Fertilizers as Strategies to Improve Crop Biomass, Yields and Physicochemical Parameters of Soil. Nutrient Dynamics for Sustainable Crop Production.

[65] Aubert G. (1978). Méthodes d’analyse Des Sols.

[66] Olsen S., Sommers L. (1982). Phosphorus. Methods of Soil Analyses, Part 2. Chemical and Microbiological Properties. Agron. Monogr..

[67] Bremner J.M. (1960). Determination of Nitrogen in Soil by the Kjeldahl Method. J. Agric. Sci..

[68] Arnon D.I. (1949). Copper Enzymes in Isolated Chloroplasts. Polyphenoloxidase in Beta Vulgaris. Plant Physiol..

[69] Savicka M., Škute N. (2010). Effects of High Temperature on Malondialdehyde Content, Superoxide Production and Growth Changes in Wheat Seedlings (*Triticum aestivum* L.). Ekologija.

[70] Aebi H. (1984). Catalase in Vitro. Methods Enzymol..

[71] DuBois M., Gilles K.A., Hamilton J.K., Rebers P.A., Smith F. (1956). Colorimetric Method for Determination of Sugars and Related Substances. Anal. Chem..

[72] Carillo P., Mastrolonardo G., Nacca F., Parisi D., Verlotta A., Fuggi A. (2008). Nitrogen Metabolism in Durum Wheat under Salinity: Accumulation of Proline and Glycine Betaine. Funct. Plant Biol..

[73] Bradford M.M. (1976). A Rapid and Sensitive Method for the Quantitation of Microgram Quantities of Protein Utilizing the Principle of Protein-Dye Binding. Anal. Biochem..

[74] Gauillard F., Richard-Forget F., Nicolas J. (1993). New Spectrophotometric Assay for Polyphenol Oxidase Activity. Anal. Biochem..

[75] Singleton V.L., Rossi J.A. (1965). Colorimetry of Total Phenolics with Phosphomolybdic-Phosphotungstic Acid Reagents. Am. J. Enol. Vitic..

[76] Al-Farsi M.A., Lee C.Y. (2008). Optimization of Phenolics and Dietary Fibre Extraction from Date Seeds. Food Chem..

[77] R Foundation for Statistical Computing (2021). A Language and Environment for Statistical Computing (4.4.0).

