# Supplementary figures and images for: Synergistic Effect of Compost and Subsurface Water Retention Technology on Optimizing Soil Properties and Argan (Argania spinosa L. Skeels) Performances Under Field Conditions
Source: Plants (Basel). 2026 Jan 24;15(3):365. doi: 10.3390/plants15030365 (PMC12899142; doi:10.3390/plants15030365)

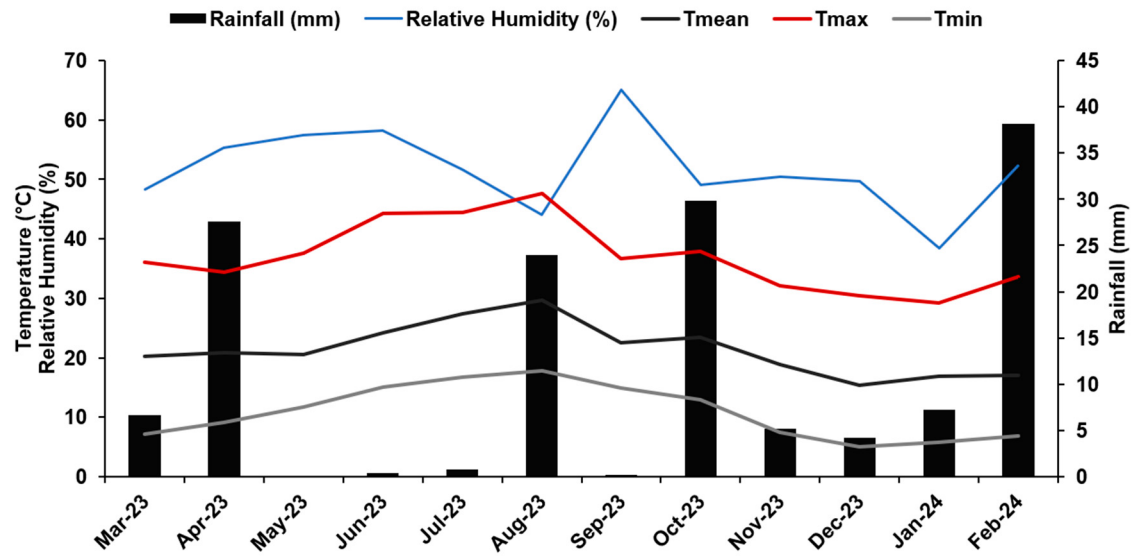

Figure S1. Meteorological data during the experiment period

Supplement: Supplementary file 1 [file plants-15-00365-s001.zip › plants-4070286-supplementary.pdf]
